# Why Do They Return? A Phenomenological Study of the Lived Experiences and Ethical Dimensions of Revision Rhinoplasty in Iran

**DOI:** 10.1111/hex.70734

**Published:** 2026-06-18

**Authors:** Habib Azimi, Zeinab Habibpour, Abbas Khalilpour, Leila Mokhtari

**Affiliations:** ^1^ Department of Otolaryngology Khoy University of Medical Sciences Khoy Iran; ^2^ Department of Nursing Khoy University of Medical Sciences Khoy Iran; ^3^ Department of Operating Room Khoy University of Medical Sciences Khoy Iran

**Keywords:** Iran, plastic surgery, qualitative research, rhinoplasty, self‐concept, treatment failure

## Abstract

**Introduction:**

Rhinoplasty is among the most common facial surgeries. Evidence indicates that the rate of revision rhinoplasty remains high, particularly in Iran. While the technical causes are well documented, the lived experiences and underlying motivations for repeated revisions remain largely unexplored. This phenomenological study aimed to explain patients' experiences and the fundamental reasons for undergoing revision rhinoplasty.

**Methodology:**

In this phenomenological study, using Colaizzi's method, participants were recruited from three teaching hospitals using purposive sampling. In‐depth, semi‐structured interviews were used for data collection and continued until data saturation. Data analysis followed Colaizzi's seven steps, utilising MAXQDA 12 software. Trustworthiness was ensured through member checking, peer debriefing, audit trail and reflexivity, in line with Lincoln and Guba's criteria. As an ethical commitment, all participants received a free clinical follow‐up by an ENT specialist. To the best of our knowledge, this is the first study in Iran to explore the psychosocial and ethical dimensions of revision rhinoplasty from the patient's perspective.

**Results:**

Analysis of 18 in‐depth interviews identified three interrelated themes crystallising around the central phenomenon: ‘Reconstructing the Self: The Search for a Lost Identity’. Participants described experiences related to: (1) A Fractured Sense of Lived Wholeness: The Struggle to Reclaim a Lost Self, marked by an identity crisis stemming from the unattainable ‘desired self’ and loss of their pre‐surgical identity; (2) Disruption of Embodied Functionality: The Severed Connection to the Corporeal Self, involving a loss of the functional self, breathing difficulties and bodily alienation; and (3) The Unravelling of the Cared‐for Self: Navigating Therapeutic Disenchantment and the Quest for Reconstruction, characterised by striving for self‐reconstruction amidst ineffective therapeutic encounters, including unrealistic promises and inadequate post‐operative support.

**Discussion:**

Revision rhinoplasty in Iran extends beyond aesthetic concerns, representing an existential quest to reconstruct a self‐fractured by profound identity loss, disrupted embodied functionality, and disillusionment with therapeutic processes. Centred on the phenomenon ‘Reconstructing the Self: The Search for a Lost Identity’, these findings underscore the critical need for ethically grounded, patient‐centred approaches. These approaches should incorporate comprehensive psychosocial support and prioritise the preservation of bodily integrity within cosmetic surgical care.

**Patient or Public Contribution:**

Participants contributed to the validation of interpreted themes through member checking. As an ethical safeguard, all participants were offered a free ENT follow‐up.

## 1 Introduction

Beauty remains a culturally ingrained and socially constructed phenomenon, defined by inherent subjectivity. The lack of a universal standard makes it a challenging variable within medical decision‐making [1]. As the focal point of the face, the nose uniquely integrates essential physiological functions with significant aesthetic value [2]. Rhinoplasty is a commonly performed procedure in aesthetic facial plastic surgery [3]. It is widely regarded as the most common and technically demanding procedure in facial plastic surgery. It aims to restore nasal function while achieving aesthetic harmony [4]. Aesthetic improvements should not compromise physiological well‐being, particularly when preserving or restoring nasal airflow, especially in cases involving obstructive conditions [5].

Although surgical techniques have advanced considerably, primary rhinoplasty continues to produce suboptimal outcomes, both functional (such as persistent nasal obstruction) and aesthetic (including discordance with patient expectations or socio‐cultural standards) [6]. Consequently, revision rhinoplasty is often required, with incidence rates reported between 5% and 21% in different populations [7]. In Iran and other middle‐income countries, many patients seek initial rhinoplasty abroad, often in countries like Turkey, the UAE or South Korea, due to lower costs or perceived expertise. These patients often travel without comprehensive surgical records, pre‐ and post‐operative photographs, or formal medical documentation. Such informational gaps significantly hinder the ethical and clinical evaluation required for revision procedures [8].

The primary driver of revision rhinoplasty, however, is often not surgical error alone but a profound mismatch between patient expectations and surgical outcomes. This discordance is frequently shaped by socio‐cultural pressures, media‐driven beauty standards, and a lack of awareness about the inherent limitations of surgical intervention [9, 10]. Patient dissatisfaction remains a leading cause of revision rhinoplasty, with studies reporting dissatisfaction rates of up to 15% [11].

Nevertheless, current scholarly work has largely centred on the anatomical, functional and technical components of revision rhinoplasty. Conversely, limited academic focus has been directed towards the personal experiences, psychological motivations and the wider socio‐cultural and ethical complexities of patients' iterative surgical trajectories. Although clinical observations at institutions such as the Khoy University of Medical Sciences reveal a notably high frequency of revision cases in Iran, the phenomenon remains unexplored through a qualitative lens. This identified research deficit warrants a phenomenological inquiry to investigate a core question: What are the lived, ethical and psychosocial factors that compel patients to opt for revision rhinoplasty, notwithstanding the considerable financial, physical and psychological burdens? Consequently, this research utilises a phenomenological methodology to identify and clarify these latent drivers among patients seeking care at medical facilities in Khoy, Iran.

## 2 Methodology

### 2.1 Study Design

This study employed a qualitative phenomenological approach using Colaizzi's method to explore the lived experiences and underlying causes of revision rhinoplasty among patients seeking care at a public teaching hospital in Khoy County, West Azerbaijan Province, Iran. Colaizzi's method provides a systematic and rigorous framework for uncovering the essential structures of lived experience [12].

### 2.2 Participant Selection and Sampling

Purposive sampling was used to recruit participants with direct experience of revision rhinoplasty. Initial participants were identified through medical records at three teaching hospitals affiliated with the Khoy University of Medical Sciences and the Social Security Organization. Following ethical approval, a PhD‐qualified psychiatric nurse (Z.H.) contacted eligible patients via telephone using documented contact information.

### 2.3 Inclusion and Exclusion Criteria

Eligibility criteria included: undergoing revision rhinoplasty within the past 5 years and readiness to share personal experiences through in‐depth interview. Exclusion criteria were death or withdrawal at any stage.

### 2.4 Data Collection

Data were collected through individual, face‐to‐face, semi‐structured, in‐depth interviews. These interviews were conducted in Persian. Participant consent was obtained prior to the interviews. The interviews were coordinated with the hospitals. Data collection took place in the educational office at the teaching hospital. Interviews were conducted during both morning and evening shifts. Researchers also covered participants' travel expenses to and from the hospital.

Interviews were audio‐recorded with written consent for transcription and analysis. Data collection continued until data saturation was achieved that defined as the point at which no new meaningful themes emerged and only redundant expressions were observed [12]. After conducting 16 interviews, no new data were extracted. However, to ensure data saturation, two additional interviews were carried out. To ensure diversity, participants varied in age, gender, socio‐economic status, and urban/rural residence. Each interview lasted 30–45 min. The interview guide included core questions:

- ‘Please describe your journey from the first time you decided to undergo revision rhinoplasty’.
- ‘What challenges, physical, psychological, or social, did you face after the procedure?’
- ‘What concerns or worries did you have following the surgery?’
- ‘When and why did you decide to undergo a second operation?’
- ‘Is there anything important about your experience that I have not asked?’

### 2.5 Clinical Follow‐Up

As an ethical commitment to participant well‐being, a free clinical evaluation by a board‐certified Ear, Nose and Throat (ENT) specialist (H.A.) was offered and conducted for all participants, addressing medical concerns and strengthening trust.

### 2.6 Data Analysis

Analysis followed the seven‐step phenomenological procedure of Colaizzi's method [12]. The seven steps are as follows:

- Familiarisation: The researcher read and re‐read the interview transcripts to gain a general understanding of the data.
- Extracting Significant Statements: Statements directly relevant to the phenomenon of revision rhinoplasty were extracted from the transcripts.
- Formulating Meanings: Each extracted statement was interpreted to identify its underlying meaning.
- Clustering Themes: Related meanings were grouped into preliminary themes.
- Developing an Exhaustive Description: The preliminary themes were elaborated and synthesised into a comprehensive description of the phenomenon, capturing its various facets.
- Producing the Fundamental Structure: A clear, explicit statement describing the core structure of the experience, the essence of the phenomenon, was developed.
- Seeking Verification (Member Checking): Findings were returned to participants for validation and feedback to ensure accuracy and authenticity.

Although Colaizzi's framework was applied descriptively, subsequent interpretive contextualisation was employed in Section Discussion, 4 to situate experiential meanings within the Iranian socio‐cultural setting, consistent with Colaizzi's final integration step.

All analyses were conducted using MAXQDA 12, a widely accepted software for qualitative data analysis in health research. Transcripts were typed verbatim within 48 h of each interview and cross‐checked against audio recordings to ensure fidelity.

### 2.7 Trustworthiness and Rigour

To ensure the credibility, transferability, dependability and confirmability of findings, Lincoln and Guba's (1986) criteria for qualitative rigour were applied [13]:

- Credibility: Credibility was enhanced through prolonged engagement with the data and peer debriefing. To minimise researcher bias, bracketing was employed by the second and corresponding authors through reflexive journaling to consciously set aside personal preconceptions during the analysis process. Furthermore, member checking was conducted by sharing the preliminary codes and interpretations with four participants to confirm that the findings accurately reflected their interview accounts.
- Transferability: Ensured by providing rich, contextual descriptions of participants, settings and procedures.
- Dependability: This was maintained through detailed documentation of all analytical steps, enabling auditability by an external researcher with a doctoral degree in nursing who is an expert in qualitative research. All steps of the data analysis process, from beginning to end, were described in detail to ensure the external audit could be performed according to these documents.
- Confirmability: Ensured by maintaining an audit trail, minimising researcher bias through reflexivity and regular peer debriefing sessions, continuous monitoring from the beginning to the end of the research, and delaying literature review until after data analysis.

## 3 Results

The study included 18 participants. All of them had nose surgery prior to revision rhinoplasty. 18 interviews were conducted and 285 codes extracted. Detailed demographic characteristics of the participants are presented in Table 1.

**Table 1:** Demographic characteristics of participants.

Participant	Gender	Age (years)	Marital status	Time between surgeries (Years)	Reason for first surgery	Educational level	Residence place
P1	Female	45	Married	8	Aesthetic	Diploma	Urban
P2	Female	46	Married	7	Aesthetic	Bachelor's degree	Rural
P3	Female	25	Single	2	Aesthetic	Diploma	Urban
P4	Female	28	Married	7	Aesthetic	Bachelor's degree	Urban
P5	Female	34	Married	9	Aesthetic	Bachelor's degree	Urban
P6	Male	22	Married	7	Aesthetic	Diploma	Urban
P7	Male	28	Married	7	Deviated septum—Aesthetic	Bachelor's degree	Urban
P8	Male	32	Married	12	Deviated septum—Aesthetic	Bachelor's degree	Urban
P9	Female	36	Married	7	Aesthetic	Diploma	Urban
P10	Male	40	Single	23	Fracture after trauma—Aesthetic	Diploma	Urban
P11	Female	23	Married	5	Aesthetic	Diploma	Urban
P12	Male	23	Single	2	Aesthetic	Diploma	Urban
P13	Female	28	Single	5	Aesthetic	Master's degree	Urban
P14	Female	33	Single	6	Deviated septum—Aesthetic	Master's degree	Rural
P15	Male	33	Single	2	Trauma‐related aesthetic	Diploma	Urban
P16	Female	35	Married	15	Aesthetic	Master's degree	Urban
P17	Female	52	Married	17	Aesthetic	Elementary education	Urban
P18	Female	32	Married	12	Deviated septum—Aesthetic	Bachelor's degree	Urban

Participants' ages ranged from 22 to 52 years, with a majority being female (*n* = 12). All 18 participants had previously undergone at least one revision rhinoplasty for aesthetic reasons, sometimes accompanied by functional indications (e.g., nasal deviation or trauma). The interval between their initial and subsequent revision surgeries varied significantly, spanning 2–23 years. Most participants resided in urban areas and possessed at least a diploma‐level education.

In this study, Colaizzi's method was used for data analysis of semi‐structured interviews. Analysis of participants' narratives yielded three overarching themes and eight sub‐themes, all converging around the central phenomenon: ‘Reconstructing the Self: The Search for a Lost Identity’. The findings underscore that revision rhinoplasty extended beyond mere aesthetic correction, serving as an existential endeavour to restore a sense of wholeness and reconnect with a lost self. These emergent themes and their sub‐themes are detailed in Table 2.

**Table 2:** Main themes, sub‐themes, representative quotes and the central phenomenon emerging from Colaizzi's phenomenological analysis.

Central phenomenon	Main themes	Sub‐themes	Representative quote (summarised)
‘**Reconstructing the Self: The Search for a Lost Identity’**	A Fractured Sense of Lived Wholeness: The Struggle to Reclaim a Lost Self	– The Fading of the Pre‐Surgical Self – Internal Alienation and the Experience of Wearing an Unfamiliar ‘Visage’ – Erosion of Existential Bonds – The Collapse of Inner Peace	*‘I felt like I was wearing a mask … “me” was gone’. (P3)*
	Disruption of Embodied Functionality: The Severed Connection to the Corporeal Self	– Loss of the Functional Self and the Inability to Reclaim Embodied Agency – The Corporeal Self as a Site of Discomfort and Alienation	‘*I feel nothing on my nose … it's as if a part of my body no longer belongs to me’. (P7)*
	The Unravelling of the Cared‐for Self: Navigating Therapeutic Disenchantment and the Quest for Reconstruction	– Unmet Therapeutic and Aesthetic Expectations Fuelled by Unrealistic Promises – Insufficient Clinical Guidance During Recovery Leading to Preventable Complications	‘*My doctor said 80% would be fixed … I realized he had little experience’. (P13)*

### 3.1 Theme 1: A Fractured Sense of Lived Wholeness: The Struggle to Reclaim a Lost Self

For many participants, nasal surgery was not merely an aesthetic intervention but a profound attempt to attain an idealised and socially accepted version of the self. When the surgical outcomes proved unsatisfactory, this ‘desired self’ remained unattainable; simultaneously, returning to the ‘former self’ was equally impossible. Participants found themselves trapped in a **liminal state**: neither who they had been, nor who they aspired to become.

This fractured sense of wholeness manifested across interrelated dimensions:

1. **The Fading of the Pre‐Surgical Self:** A loss of familiarity and continuity with their prior identity.
2. **Internal Alienation and the Experience of Wearing an Unfamiliar ‘Visage’:** A sense of inauthenticity and detachment from their true self, as if wearing a mask.
3. **Erosion of Existential Bonds:** Weakening of an individual's standing in social and familial relationships, accompanied by social judgement and stigma.
4. **The Collapse of Inner Peace:** Disruption of psychological equilibrium, leading to profound hesitation and anxiety.

One participant articulated this experience:

I thought a small surgery would fix everything…. But when the bandages were removed and I looked in the mirror, my heart stopped. My nose was smaller but it felt tight, rigid, as if I were wearing a mask…. I couldn't be at ease with my family anymore. My mother said, “Oh my, you've changed so much,” and I just smiled but inside, I wanted to cry. It was as if I'd put on a mask, and “me” was gone.

P. 3

Participants also encountered intense social judgement and reactive stigma, which deepened feelings of shame and exclusion. Even among close family members, painful comparisons exacerbated this alienation:

Everyone stared at my deformed nose…. I felt like I had leprosy. Wherever I went, people asked, “Why did your nose turn out like this?”

P. 10

They mocked me…. My mother and sister kept saying, “You were prettier before.” I truly couldn't bear having this nose on my face anymore.

P. 12

At the deepest level, this identity crisis eroded psychological stability. Self‐confidence deteriorated severely, and many participants experienced profound hesitation about pursuing revision surgery, fearing further failure:

My self‐trust was shattered…. I was terrified to decide on another surgery. My spirit was gone; I wasn't the same person anymore.

P. 10

In response to this existential suffering, many were driven not towards aesthetic perfection but towards a renewed effort—an effort not to become ‘beautiful’ but to reclaim a sense of wholeness and belonging to the self.

My perfectionism went beyond reason…. I constantly thought about revision surgery. I wanted my nose to be more beautiful—so I could finally feel, once again, that I am me.

P. 18

### 3.2 Theme 2: Disruption of Embodied Functionality: The Severed Connection to the Corporeal Self

The experience of undergoing cosmetic rhinoplasty profoundly disrupted participants' sense of embodied functionality, severing their connection to their corporeal self. This disruption manifested across several dimensions, fundamentally altering their lived experience of their physical being and their interaction with the world. Two primary dimensions emerged from the data, reflecting the multifaceted nature of this disruption:

1. **Loss of the Functional Self and the Inability to Reclaim Embodied Agency:** Participants described a profound sense of losing their ‘functional self’—the self that could engage seamlessly with the physical world. This loss was characterised by an inability to perform everyday activities without conscious effort or discomfort, leading to a diminished capacity for spontaneous action and a feeling of being disconnected from their own bodies.
  I used to be so active, hiking and dancing. Now, even simple tasks feel exhausting. It's like my body is working against me.
  P. 4
  It's not just about the aesthetic; it's about feeling capable. I can't even smile properly without feeling a pull, a constant reminder that something is wrong.
  P. 7
2. **The Corporeal Self as a Site of Discomfort and Alienation:** The physical body transformed from a source of natural being into a site of discomfort, pain and alienation. Participants experienced a growing disconnect between their internal sense of self and their physical presentation, leading to feelings of being estranged from their own flesh.

There's this constant dull ache, a tightness I can't shake off. It's like my face is a mask I'm forced to wear, and it's not really mine.

P. 1

I feel … foreign in my own skin. The swelling and bruising went away, but this feeling of disconnection, of my body being something separate, remains.

P. 13

This disruption of embodied functionality and the resulting alienation from the corporeal self‐underscored the deep psychological and existential impact of cosmetic rhinoplasty when it fails to align with the individual's lived sense of being.

### 3.3 Theme 3: The Unravelling of the Cared‐for Self: Navigating Therapeutic Disenchantment and the Quest for Reconstruction

This theme captures the profound sense of disillusionment and unravelling experienced by participants due to ineffective therapeutic encounters, particularly concerning their cosmetic rhinoplasty journey. A core concept here is the failure of the healthcare system to provide adequate care, leaving individuals feeling abandoned and hindering their pursuit of self‐reconstruction. This theme unfolds across two primary dimensions:

1. **Unmet Therapeutic and Aesthetic Expectations Fuelled by Unrealistic Promises:** Participants highlighted how their expectations for therapeutic and aesthetic outcomes were severely undermined by unrealistic promises made by their surgeon. This gap between promised results and actual outcomes led to significant disappointment and a sense of betrayal.
  He promised me a perfect nose, a complete transformation. But what I got was … different. It's not what we discussed, and now I feel stuck with it.
  P. 4
  The surgeon showed me before‐and‐after pictures, all perfect. But my recovery was full of complications, and the final result doesn't match those initial promises at all.
  P. 13
2. **Insufficient Clinical Guidance During Recovery Leading to Preventable Complications:** A critical aspect of the therapeutic disenchantment was the lack of adequate clinical guidance and support during the crucial post‐operative recovery period. This deficiency resulted in preventable complications and exacerbated the participants' feelings of being uncared for.

Nobody really told me what to expect after the surgery. I was sent home with minimal instructions, and when problems started, it was hard to get clear advice.

P. 1

I had persistent swelling and pain for months. I called the clinic multiple times, but they brushed it off, saying it was normal. If they had given me better post‐op care instructions, maybe this wouldn't have happened.

P. 2

The unravelling of the ‘cared‐for self’ in Theme 3 underscores the critical importance of realistic patient–provider communication, thorough pre‐operative counselling and robust post‐operative support in the context of cosmetic procedures. The quest for self‐reconstruction is significantly hampered when the therapeutic journey itself becomes a source of distress and disillusionment.

## 4 Discussion

The findings of this study suggest that, for many patients, rhinoplasty was perceived as far more than a cosmetic procedure; rather, it was an attempt to restore a sense of self that they felt had been diminished or lost. When the surgical result did not correspond with their internal image of the desired self, participants described experiencing a disruption in their sense of identity and personal continuity. Many felt that they were no longer fully aligned with who they had been before surgery, yet had not developed a coherent post‐surgical self. This disconnect went beyond aesthetic disappointment and often evolved into a deeper identity‐related distress. Narratives such as ‘wearing a mask’, ‘feeling that the self had disappeared’, and experiencing a decline in social and familial status reflect this broader struggle for self‐reconstruction in the context of disrupted identity.

These experiences were notably magnified within the specific socio‐cultural landscape of Iran. Unlike Western research, which frequently categorises aesthetic surgery dissatisfaction as a product of ‘unrealistic expectations’ or ‘individual perfectionism’ [14], our findings indicate that familial and social pressures are paramount; for instance, relatives' persistent focus on pre‐surgical features often exacerbated patients' feelings of self‐erasure. This observation aligns with evidence from Zare and Sadeghi (2021), who reported that rather than enhancing psychological well‐being, cosmetic interventions may deepen identity crises in those predisposed to feelings of inferiority or diminished social standing, especially when surgical outcomes diverge from the patient's internal expectations [15].

These internal pressures were further aggravated by the constant exposure to digitally altered beauty standards disseminated across mass and social media, which often present unrealistic ideals. Consequently, for many participants, the motivation for surgery was not merely the attainment of ‘beauty’ but rather the recovery of a perceived lost self. This perspective significantly diverges from individualistic Western models, which generally frame cosmetic surgery as a purely autonomous, self‐directed choice [16]. In contrast, within collectivist settings like Iran, aesthetic standards are deeply embedded as social capital; thus, discontent with one's physical appearance is often experienced as a broader threat to one's social identity and interpersonal familial bonds.

These experiences are further substantiated by a systematic review from Katamanin et al. (2024), which indicates that post‐cosmetic surgery dissatisfaction across diverse societies stems from a systemic disruption of self‐identity and social belonging, rather than solely personal expectations [17]. This aligns precisely with our participants' narratives of ‘self‐annihilation’ and ‘feeling lost’. Furthermore, research by Farzan et al. (2025) confirms that dissatisfaction following rhinoplasty leads to a significant worsening of anxiety and depression, underscoring that this crisis is a profound psychological and identity‐based disturbance, particularly intensified in societies like Iran by social and familial pressures [18].

For these patients, rhinoplasty was not simply a cosmetic procedure; it was closely linked to respiratory function and the experience of bodily wholeness. After surgery, the nose—previously perceived as a natural and unnoticed component of breathing—no longer performed its essential role. Beyond physical discomfort, this loss led participants to experience a diminished sense of control over their bodily existence, as though they were no longer living in a state of effortless and unremarkable embodiment. This experience appeared in two related forms: first, an inability to breathe freely; and second, a broader disconnection from the body, in which patients no longer felt familiarity with or ownership over their bodily selves. This finding is consistent with the notion of ‘identity disruption’ after facial alteration, in which the body is no longer experienced as a familiar or trustworthy home [19]. Moreover, such functional problems—particularly respiratory difficulties—are often insufficiently addressed by clinical teams, leaving patients feeling marginalised and unrecognised [20]. In revision rhinoplasty, this experience becomes even more pronounced: patients are not only distressed by unmet aesthetic goals but also deprived of a basic function they had previously taken for granted [21]. In this sense, respiratory dysfunction is not merely a surgical complication; it may also be experienced as a profound loss of self.

Post‐surgical dysfunction after rhinoplasty was not viewed solely as a technical error; rather, it was perceived as a consequence of systemic shortcomings within the care trajectory. Participants indicated that unrealistic or poorly articulated expectations, initially set by their surgeons, remained unaddressed, leaving them without adequate support during crucial recovery periods. This sense of being ‘abandoned’ cultivated deep feelings of isolation and helplessness, especially when patients attempted to seek professional guidance or clinical reassurance regarding emerging symptoms. Such experiences are consistent with evidence suggesting that functional impairments post aesthetic surgery are frequently disregarded during follow‐up assessments, resulting in patient feelings of neglect [20]. This phenomenon manifested in two distinct ways: first, the failure of therapeutic outcomes to meet what were often unsubstantiated surgical assurances; and second, the deficiency in comprehensive pre‐ and post‐operative education, which might have mitigated preventable complications. Ultimately, these shortcomings were interpreted not merely as operational lapses but as ethical failures in clinical duty, where patients were reduced to surgical ‘cases’ rather than treated as individuals. This perspective reflects broader findings where informed consent is often reduced to a procedural formality devoid of moral substance [22] and where institutional indifference serves to marginalise the patient's voice [23].

This study was conducted in a specific geographic and cultural context (Khoy, Iran), which may limit the transferability of findings to other socio‐cultural settings. Although purposive sampling ensured diversity in age, gender and residence, participants were recruited from teaching hospitals, potentially excluding those who sought care in private clinics in other cities. The participants' recruitment technique in this study may have introduced selection bias, as patients receiving care outside of academic centres were excluded. This could also limit the generalisability of the data. Additionally, recall bias may have influenced narratives, as some participants reflected on surgeries performed up to 5 years prior. Finally, while MAXQDA facilitated systematic coding, the interpretive nature of phenomenology inherently involves researcher subjectivity, mitigated here through reflexivity and audit trails, but not eliminable.

Given these findings, we recommend integrating psychosocial assessment and realistic expectation‐setting into pre‐operative consultations, involving mental health professionals in cosmetic surgery pathways, and establishing mandatory post‐operative support systems—including accessible ENT specialist follow‐up and structured patient education. Regulatory bodies in Iran should also consider refining informed consent protocols to explicitly address risks related to identity disruption, functional impairment and potential need for revision. Future research could explore longitudinal trajectories or compare experiences across care settings, including foreign patients in the medical tourism system.

## 5 Conclusion

This study reveals that revision rhinoplasty in the Iranian context transcends aesthetic correction and functions as an existential quest to reconstruct a fragmented self. Participants' decisions to undergo repeated surgeries were driven not by superficial vanity but by profound disruptions in identity, bodily integrity and perceived failures in the therapeutic process. The central phenomenon ‘Reconstructing the Self: The Search for a Lost Identity’ captures how patients navigate a liminal space between a lost former self and an unattained ideal, while also confronting functional impairment and systemic shortcomings in care. Acknowledging the study's limitations, particularly its specific cultural context and sampling methods, these findings strongly underscore the necessity of repositioning revision rhinoplasty not merely as a surgical challenge but as a psychosocial and ethical phenomenon. Therefore, holistic, patient‐centred approaches, including enhanced pre‐operative psychosocial assessments, robust post‐operative support systems and refined informed consent protocols addressing identity and functional concerns, are crucial for both clinical practice and future research endeavours.

## Author Contributions

**Habib Azimi:** conceptualisation, supervision, investigation, writing – review and editing. **Zeinab Habibpour:** software, data curation, formal analysis, validation, resources, writing – original draft, writing – review and editing. **Abbas Khalilpour:** methodology, data curation, writing – original draft, writing – review and editing. **Leila Mokhtari:** conceptualisation, methodology, software, data curation, investigation, validation, formal analysis, supervision, resources, project administration, visualisation, funding acquisition, writing – original draft, writing – review and editing.

## Ethics Statement

The study received ethical approval from the Research Ethics Committee of Khoy University of Medical Sciences (Approval Code: IR.KHOY.REC.1403.014, approval date June 2, 2024). Confidentiality was strictly maintained: all identifiers were removed from transcripts, and audio files were stored securely. Participants were informed of their right to withdraw at any time without consequence. The free clinical evaluation provided as part of the study further reinforced our commitment to ethical responsibility and participant welfare.

## Consent

Written informed consent was obtained from all participants prior to interviews.

## Conflicts of Interest

The authors declare no conflicts of interest.

## Consent for Publication

Not applicable.

## Data Availability

The data that support the findings of this study are available on request from the corresponding author. The data are not publicly available due to privacy or ethical restrictions.
